# Diagnostic performance and reader confidence of MRI-derived CT-like images in patients with femoral head necrosis

**DOI:** 10.1186/s41747-026-00714-1

**Published:** 2026-04-21

**Authors:** Felix L. Herr, Natascha Hohmann, Christian Dascalescu, Veronika Gottfried, Markus Bormann, Boj F. Hoppe, Lennart Schröder, Jens Ricke, Boris M. Holzapfel, Alexandra Gersing, Nina Hesse, Jörg Arnholdt, Paul Reidler

**Affiliations:** 1 Department of Radiology, University Hospital, LMU Munich, Munich, Germany; 2 Department of Orthopaedics and Trauma Surgery, Orthopaedic Oncology, Musculoskeletal University Center Munich (MUM), University Hospital, LMU Munich, Munich, Germany; 3 Department of Neuroradiology, University Hospital, LMU Munich, Munich, Germany; 4 Department of Orthopedics and Traumatology, Paracelsus Medical University, 90741 Nuremberg, Germany

**Keywords:** Femur head necrosis, Magnetic resonance imaging, Radiography, Reproducibility of results, Tomography (X-ray computed)

## Abstract

**Abstract:**

Femoral head necrosis (FHN) requires accurate imaging for staging and treatment planning. While MRI is sensitive, conventional sequences may miss subchondral fractures. A CT-like MRI sequence combines CT-like structural detail with soft-tissue contrast. We evaluated its performance for Association Research Circulation Osseous (ARCO) staging compared with MRI and radiography. In this retrospective study, 21 patients (33 hips) with confirmed FHN underwent radiography and MRI, including T1- and T2-weighted sequences and a T1-weighted gradient-echo CT-like sequence. Two musculoskeletal imaging specialists assessed ARCO stage, reader confidence, and image quality. Interrater reliability was evaluated using the intraclass correlation coefficient, intermodality agreement with weighted Cohen’s *κ*, and modality comparisons with Wilcoxon signed-rank tests. CT-like MRI demonstrated higher ARCO staging than radiography (*p* = 0.004), T1-weighted (*p* = 0.031), and T2-weighted MRI (*p* = 0.046). Upstaging compared with conventional modalities occurred in 6 of 33 hips (18.2%). Reader confidence was highest for CT-like MRI (all *p* ≤ 0.003), and image quality was superior compared with T1- and T2-weighted MRI (both *p* < 0.001). Agreement in ARCO staging was excellent with T1-weighted and T2-weighted images and substantial with radiography. CT-like MRI improved image quality and diagnostic confidence, enabling more accurate FHN assessment and detection of subchondral collapse missed by conventional MRI.

****Relevance statement**:**

CT-like MRI improves detection of early femoral head collapse, enabling more accurate staging and treatment planning.

****Key Points**:**

- Femoral head necrosis requires precise imaging to accurately stage the disease.
- Conventional MRI may miss early subchondral femoral head fractures.
- CT-like MRI enhances visualization of bone microstructure, diagnostic confidence and image quality.
- Improved staging with CT-like MRI may influence clinical management.

**Graphical Abstract:**

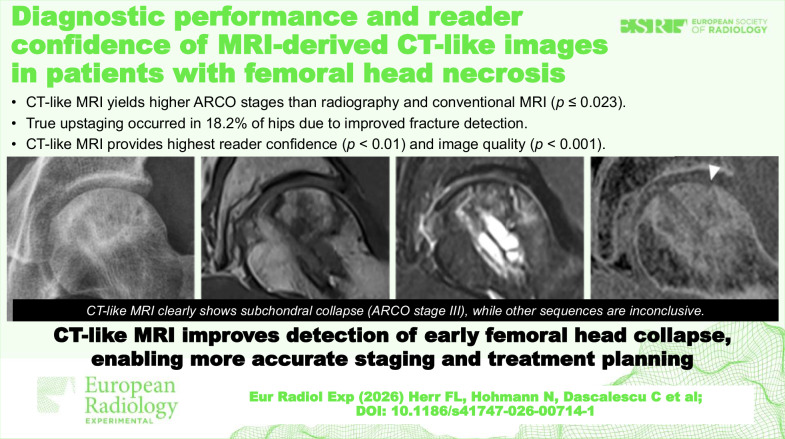

## Background

Femoral head necrosis (FHN) is caused by impaired vascular supply and may progress to subchondral fracture and collapse of the articular surface [1]. Imaging is central to diagnosis and staging. Radiography is typically the first-line modality but is often normal in early disease, with sclerosis, cystic changes, or the “crescent sign” appearing only at advanced stages [2, 3]. Computed tomography (CT) reliably detects subchondral fractures and collapse but involves ionizing radiation and is usually reserved for inconclusive cases [2, 3]. Magnetic resonance imaging (MRI) remains the most sensitive modality for early FHN detection, enabling visualization of bone marrow edema and necrosis [1]. Disease severity is classified using the Association Research Circulation Osseous (ARCO) system [4]. Although the updated 2019 ARCO criteria no longer require routine CT [5], conventional MRI has limited ability to depict calcified cortical bone [3]. This has driven interest in advanced “CT-like” MRI techniques, including ultrashort echo time and high-resolution gradient-echo sequences, which provide detailed bone visualization without radiation exposure [6–8].

In the context of FHN, improved depiction of subtle subchondral fractures may enable earlier detection of collapse and more precise ARCO staging. This could increase radiologists’ diagnostic confidence and reduce understaging. Although early evidence is promising, the routine clinical value of CT-like MRI for FHN assessment remains uncertain. We hypothesized that CT-like MRI improves diagnostic accuracy and reader confidence for ARCO staging compared with conventional MRI sequences and radiography. The aim of this study was to validate this approach in a clinical cohort.

## Materials and methods

### Study population

This retrospective study included all consecutive patients who underwent a dedicated CT-like MRI protocol for suspected FHN at our institution between 2022 and 2024. Inclusion criteria were the availability of CT-like MRI together with all corresponding conventional imaging modalities, including radiography and T1- and T2-weighted MRI. Exclusion criteria comprised incomplete imaging datasets or insufficient image quality precluding reliable evaluation.

### Imaging protocol

The CT-like sequence was implemented as part of the standard imaging protocol and optimized for cortical bone visualization. MRI was performed on a 3-T clinical scanner (MAGNETOM Skyra, Siemens Healthineers). A T1-weighted three-dimensional gradient-echo sequence was used with the following parameters: echo time 2.01 ms; repetition time 5.3 ms; flip angle 8°; field of view 375 × 250 mm²; matrix 384 × 256; isotropic voxel size 0.5 × 0.5 × 0.5 mm³; acquisition time 4:24 min. The sequence was acquired in coronal orientation and reformatted in coronal, axial, and sagittal planes with a slice thickness of 1.0 mm.

### Rating system

All imaging datasets were independently reviewed by two experienced board-certified specialists (one radiologist and one orthopedic surgeon), each with approximately 10 years of musculoskeletal imaging experience, to ensure a high level of diagnostic expertise. All modalities (CT-like MRI, T1-weighted MRI, T2-weighted MRI, and radiography) were evaluated separately in randomized order. Readers were blinded to clinical information, prior or follow-up imaging, and each other’s assessments.

Image analysis included assessment of ARCO stage (I–IV), reader confidence, and image quality for each modality. Reader confidence and image quality were rated using 5-point Likert scales, where 1 indicated high confidence or excellent image quality and 5 indicated low confidence or poor image quality. Interrater reliability for ARCO staging, reader confidence, and image quality was assessed using intraclass correlation coefficients (ICC) based on a two-way random-effects model for absolute agreement with single measurements, as recommended by Koo and Li [9]. ICC values were interpreted as poor (< 0.50), moderate (0.50–0.75), good (0.75–0.90), or excellent (> 0.90). For intermodality comparisons, mean values of both readers were used. Agreement between CT-like MRI and conventional imaging modalities was assessed using weighted Cohen’s *κ* with squared weights [10]. Each imaging modality was evaluated independently and separately for each hip. No external reference standard was available. Therefore, analyses focused on intermodality differences in ARCO staging rather than diagnostic accuracy metrics.

### Statistical analysis

ARCO stages, reader confidence, and image quality were summarized as median and interquartile range (IQR) due to their ordinal nature. Comparisons between CT-like MRI and reference modalities were performed using paired Wilcoxon signed-rank tests, with *p*-values adjusted for multiple comparisons using the Holm–Bonferroni method. An adjusted *p*-value < 0.05 was considered statistically significant.

All consecutive patients who met the inclusion criteria and underwent the dedicated CT-like MRI protocol for suspected FHN during the study period were included; therefore, no a priori sample size calculation was performed. Statistical analyses were conducted using R (version 4.3.0, R Foundation for Statistical Computing).

## Results

Twenty-one patients (33 hips) with confirmed FHN were included. The cohort comprised 15 men and 6 women, aged 39.2 ± 14.5 years (mean ± standard deviation).

### ARCO classification

**Fig. 1:**
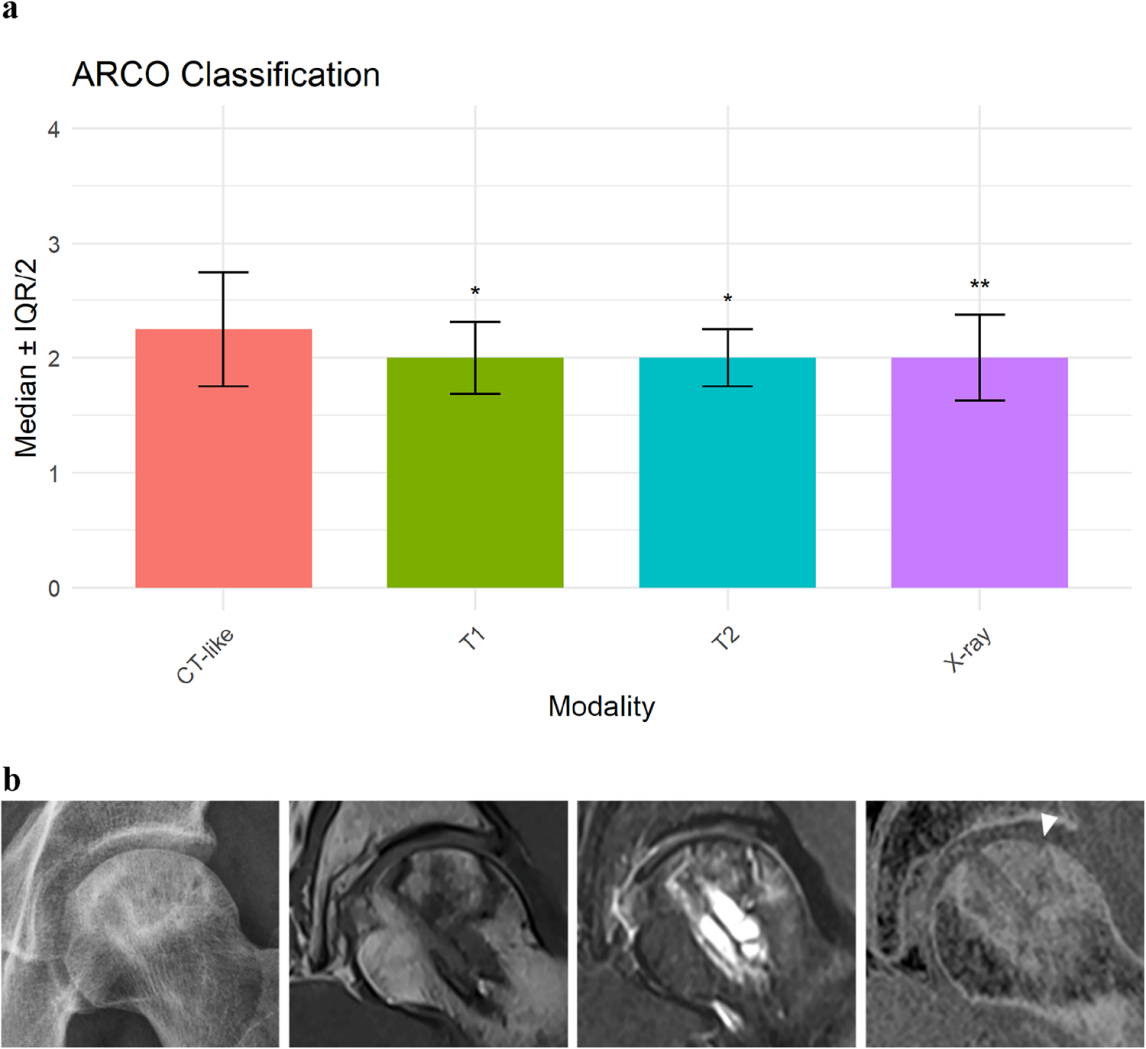
ARCO classification by imaging modality and patient example. **a** Bar chart illustrating median ARCO classification scores (with interquartile ranges) across four imaging modalities. The CT-like MRI sequence (red bar) demonstrated a higher median ARCO stage (median 2.25, IQR 1.0) than radiography (purple bar; median 2.00, IQR 0.75; adjusted *p* = 0.004), T1-weighted MRI (green bar; median 2.00, IQR 0.625; adjusted *p* = 0.031), and T2-weighted MRI (cyan bar; median 2.00, IQR 0.50; adjusted *p* = 0.046). Although median values appeared similar, CT-like MRI consistently yielded higher staging results. Asterisks above the bars indicate statistically significant differences compared with the CT-like sequence (adjusted *p < 0.05, **p < 0.01). **b** A 42-year-old man with femoral head necrosis 16 months after core decompression. From left to right: X-ray, conventional T1-weighted, T2-weighted short tau inversion-recovery (STIR), and inverted T1 gradient-echo CT-like images. The CT-like sequence clearly depicts subchondral bone collapse (ARCO stage III, arrowhead), while other sequences remain ambiguous. ARCO Association Research Circulation Osseous

CT-like MRI demonstrated a higher median ARCO stage (median 2.25, IQR 1.0) than radiography (median 2.00, IQR 0.75; adjusted *p* = 0.004), T1-weighted MRI (median 2.00, IQR 0.625; adjusted *p* = 0.031), and T2-weighted MRI (median 2.00, IQR 0.50; adjusted *p* = 0.046). Although median values appeared similar, CT-like MRI resulted in higher ARCO staging in 6 of 33 hips (18.2%), reflecting detection of early subchondral collapse not identified on conventional imaging (Fig. 1a). An illustrative example is shown in Fig. 1b. Agreement in ARCO classification was excellent between CT-like MRI and T1-weighted MRI (*κ* = 0.904) and T2-weighted MRI (*κ* = 0.869), and substantial between CT-like MRI and radiography (*κ* = 0.709). Interrater reliability for ARCO classification was good to excellent across all modalities (ICC 0.82–0.93).

### Reader confidence

**Fig. 2:**
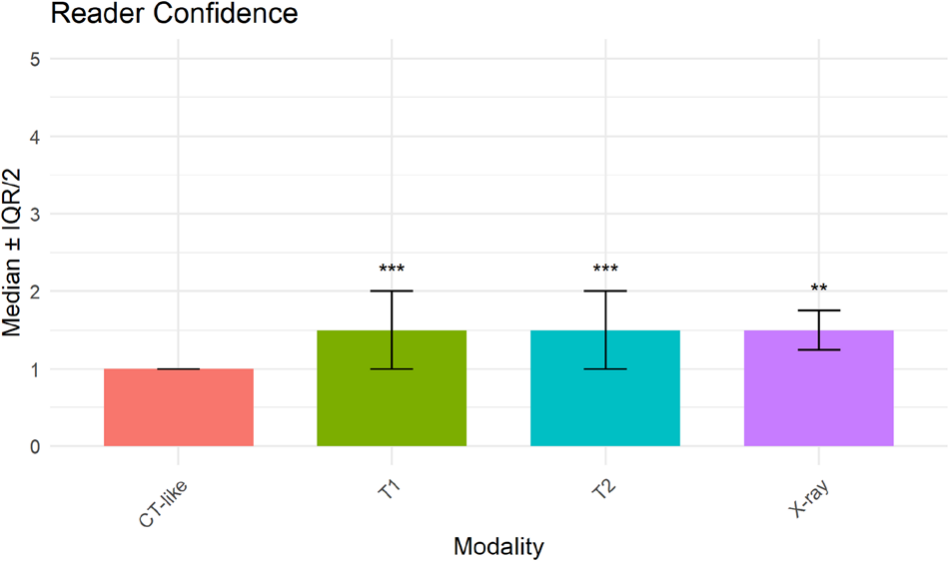
Reader confidence by imaging modality. Bar chart illustrating median reader confidence scores (with interquartile ranges) across four imaging modalities. Lower values indicate higher diagnostic confidence (1 = high, 5 = low). The CT-like MRI sequence (red bar) demonstrated the highest diagnostic confidence (median 1.0, IQR 0), compared with T1-weighted MRI (green bar; median 1.5, IQR 1.0; adjusted *p* < 0.001), T2-weighted MRI (cyan bar; median 1.5, IQR 1.0; adjusted *p* < 0.001), and radiography (purple bar; median 1.5, IQR 0.5; adjusted *p* = 0.003). Asterisks above the bars indicate statistically significant differences compared with the CT-like sequence (adjusted **p < 0.01, ***p < 0.001)

Diagnostic confidence was highest for CT-like MRI (median 1.0, IQR 0), compared with T1-weighted MRI (median 1.5, IQR 1.0; adjusted *p* < 0.001), T2-weighted MRI (median 1.5, IQR 1.0; adjusted *p* < 0.001), and radiography (median 1.5, IQR 0.5; adjusted *p* = 0.003; Fig. 2). All differences were statistically significant (*p* < 0.010). Interrater agreement for confidence ratings was low across modalities (ICC < 0.40).

### Image quality

**Fig. 3:**
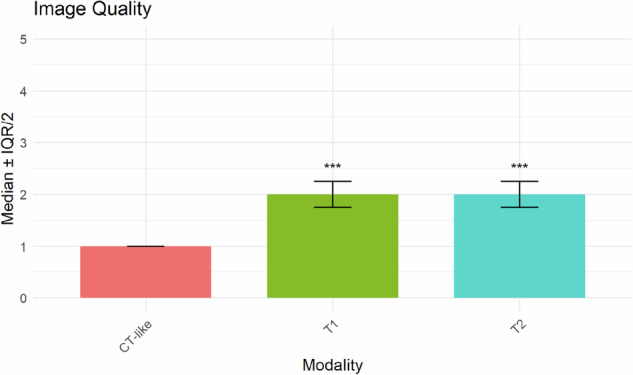
Image quality by imaging modality. Bar chart illustrating median image quality scores (with interquartile ranges) for three MRI modalities. Lower values indicate better perceived image quality (1 = good, 5 = poor). The CT-like MRI sequence (red bar) demonstrated superior image quality (median 1.0, IQR 0) compared with T1-weighted MRI (green bar; median 2.0, IQR 0.5; adjusted *p* < 0.001) and T2-weighted MRI (cyan bar; median 2.0, IQR 0.5; adjusted *p* < 0.001). Asterisks above the bars indicate statistically significant differences compared with the CT-like sequence (adjusted ***p < 0.001). Radiography was not included, as image quality was not assessed for X-ray in this study

CT-like MRI achieved superior image quality (median 1.0, IQR 0), compared with T1-weighted MRI (median 2.0, IQR 0.5; adjusted *p* < 0.001) and T2-weighted MRI (median 2.0, IQR 0.5; adjusted *p* < 0.001; Fig. 3). Interrater reliability for image quality was highest for CT-like MRI (ICC = 0.84) and substantially lower for conventional sequences (T1-weighted: ICC = 0.37; T2-weighted: ICC = 0.29).

## Discussion

The ability of CT-like MRI to depict cortical bone has already been demonstrated in other regions. In cochlear implantation planning, CT-like MRI provided reliable visualization of the temporal bone and sufficient information for surgery, suggesting it may reduce the need for diagnostic CT [11]. Similarly, for hand and wrist fractures, CT-like MRI showed substantial agreement with CT, supporting its role as a radiation-free alternative for cortical bone assessment [7]. This study demonstrates that CT-like MRI more frequently identifies higher ARCO FHN stages compared with conventional MRI and radiography. Even small differences in staging may be clinically relevant. ARCO stage III, defined by subchondral fracture, represents a critical threshold beyond which joint-preserving treatments are generally no longer feasible [5]. In contrast, stage II disease may still be treated with procedures such as core decompression or biologic augmentation [12].

Although conventional MRI is highly sensitive for early FHN, it may miss subtle cortical disruptions, leading to understaging [13]. Previous studies reported MRI sensitivities as low as 38% for detecting subchondral fractures, whereas CT achieved near-complete detection [14]. In our cohort, CT-like MRI improved visualization of cortical bone and subchondral fractures, resulting in higher ARCO stages and upstaging in 18.2% of hips. Agreement between CT-like MRI and T1- and T2-weighted MRI was excellent, while agreement with radiography was lower, reflecting radiography’s limited sensitivity. Interobserver reliability for ARCO staging was high and exceeded values reported in earlier ARCO studies [15]. Reader confidence was highest for CT-like MRI, consistent with previous musculoskeletal studies using CT-like MRI techniques such as zero-echo time [16]. Although confidence ratings showed low interrater agreement, this likely reflects the subjective nature of confidence assessment rather than limitations of the technique itself [14, 17]. Image quality ratings favored CT-like MRI and showed high interrater agreement, consistent with prior studies of ultrashort echo time‒UTE and zero echo time techniques [17, 18].

Limitations include the retrospective, single-center design and small sample size. The limited sample size reflects the exploratory nature of this study and the restricted availability of the dedicated CT-like MRI protocol during the study period. Larger, prospective multicenter studies are warranted to confirm these findings and to assess their generalizability. Another limitation is the absence of an external reference standard, precluding assessment of diagnostic accuracy and limiting external validation, although the ARCO 2019 criteria no longer mandate CT for routine staging [5]. Availability of CT-like sequences may also limit widespread implementation. Including bilateral hips may have introduced within-patient dependence. However, a sensitivity analysis using one randomly selected hip per patient yielded qualitatively consistent interrater reliability estimates, supporting robustness of the findings.

In conclusion, CT-like MRI improves assessment of femoral head necrosis by providing higher ARCO stages, superior image quality, and greater diagnostic confidence compared with conventional MRI and radiography. This or similar techniques have the potential to complement traditional MRI and replace diagnostic CT in FHN, allowing accurate one-step MRI staging of disease and facilitating timely, appropriate management while avoiding unnecessary radiation exposure.

## Data Availability

The datasets used and/or analyzed during the current study are available from the corresponding author on reasonable request.

## Abbreviations

ARCO: Association Research Circulation Osseous
CT: Computed tomography
FHN: Femoral head necrosis
ICC: Intraclass correlation coefficient
MRI: Magnetic resonance imaging
